# Metadynamics Simulations Reveal a Na^+^ Independent Exiting Path of Galactose for the Inward-Facing Conformation of vSGLT

**DOI:** 10.1371/journal.pcbi.1004017

**Published:** 2014-12-18

**Authors:** Ina Bisha, Alex Rodriguez, Alessandro Laio, Alessandra Magistrato

**Affiliations:** 1 SISSA, Trieste, Italy; 2 CNR-IOM-Democritos National Simulation Center, SISSA, Trieste, Italy; Baltimore, United States of America

## Abstract

Sodium-Galactose Transporter (SGLT) is a secondary active symporter which accumulates sugars into cells by using the electrochemical gradient of Na^+^ across the membrane. Previous computational studies provided insights into the release process of the two ligands (galactose and sodium ion) into the cytoplasm from the inward-facing conformation of *Vibrio parahaemolyticus* sodium/galactose transporter (vSGLT). Several aspects of the transport mechanism of this symporter remain to be clarified: (i) a detailed kinetic and thermodynamic characterization of the exit path of the two ligands is still lacking; (ii) contradictory conclusions have been drawn concerning the gating role of Y263; (iii) the role of Na^+^ in modulating the release path of galactose is not clear. In this work, we use bias-exchange metadynamics simulations to characterize the free energy profile of the galactose and Na^+^ release processes toward the intracellular side. Surprisingly, we find that the exit of Na^+^ and galactose is non-concerted as the cooperativity between the two ligands is associated to a transition that is not rate limiting. The dissociation barriers are of the order of 11–12 kcal/mol for both the ion and the substrate, in line with kinetic information concerning this type of transporters. On the basis of these results we propose a branched six-state alternating access mechanism, which may be shared also by other members of the LeuT-fold transporters.

## Author Summary

Membrane proteins are crucial for the communication of the cell with the environment. Among these, symporters are in charge of the transport of molecules (like sugars, amino acids, osmolytes) inside the cells, exploiting the concentration gradient of an ion to perform the task. Here we investigate by atomistic simulations the transport mechanism of the Sodium-Galactose symporter. Our results allow constructing a detailed and quantitative model of the release process of the two ligands. Surprisingly, we find that the galactose is released to the cytosol independently from the ion, unambiguously indicating that the coupling in their transport mechanism is associated to the steps preceding the release process. A large family of symporters shares the same fold and potentially the same transport mechanism. As such our results are important also because they can provide insights on common mechanistic features of these transporters.

## Introduction

Secondary active transporters are membrane proteins involved in the translocation of small organic molecules across the cellular membrane using the energy stored as transmembrane electrochemical gradient of ions (mostly Na^+^ or H^+^). Sodium symporters in particular use this alkali metal ion to cotransport a variety of substrates (sugars, amino acids, neurotransmitters, nucleobases) against their chemical concentration [1], [2]. These symporters share a common structural core domain called ‘LeuT-fold’ [3]–[9] and they play a crucial role in the physiology of the brain, intestine, kidney, thyroid and skin, representing thus the target for therapeutic intervention in the treatment of depression, diabetes, obesity, etc [1], [2]. The proposed transport mechanism of this kind of secondary active transporters is called ‘alternating access mechanism’ [10]. According to this mechanism, the symporters undergo a large conformational change switching from an outward-facing conformation, where the ligands bind from the extracellular medium, to an inward-facing conformation, where the ligands are released into the cytosol.

In this work we focused on the Sodium-Galactose Transporter (SGLT), a sodium symporter that accumulates sugars, like glucose or galactose, into cells. In humans this process is very important for a correct intestinal absorption and renal reabsorption, and it is nowadays a promising field for the development of a new class of drugs for the treatment of type 2 diabetes [11]. For *Vibrio parahaemolyticus* the crystal structure of the inward-facing conformation of the bacterial homologue of SGLT (vSGLT) was solved by Faham et al. [6] with galactose (Gal) bound inside the protein. While in the human transporter the substrate transport is driven by two Na^+^ ions, only one ion is required in the bacterial homologue. Yet, the Na^+^ was not solved in the X-ray structure (PDB 3DH4) and a plausible ion-binding site, corresponding to Na2 site, was proposed, on the basis of a structural comparison with the LeuT structure [4] and by mutational analysis. Subsequent, molecular dynamics (MD) simulations studies suggested that this crystal structure represents an ion-releasing state of the transporter [12]–[14]. Thus, in a previous work, using MD and metadynamics (MTD) simulations, we identified a possible ion-retaining state of the vSGLT [15].

The dissociation mechanism of galactose has also been investigated by molecular simulations studies which showed that Gal release occurs either spontaneously or by applying an external force [13], [14], [16]. These studies lead to contradictory conclusions on a possible gating role of Y263, on the exact conformational state of the transporter (open or occluded) captured crystallographically and on the free energy profile of Gal release [13], [14], [16]. Namely, Zomot et al. showed that Gal exited the protein only by using steered molecular dynamics (SMD), after the rotameric transition of the side-chain of Y263, which, according to this study, acts as a gate. A second gate represented by Y269 was also encountered later on along the path [13]. Consistently with these findings, Watanabe et al. showed that the sodium exit triggers the substrate release after the new rotameric conformation acquired by Y263 and that Gal has to overcome very small barriers (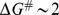 kcal/mol) along its exit pathway [14]. A different scenario was instead provided by Li and Tajkhorshid in 2011. By combining MD and SMD simulations, they identified a curved translocation pathway for Gal release. In this path Gal moves around Y263, requiring no gating event. This study pointed out that the crystal structure represents an open state of the transporter [16]. Unfortunately, experiments do not help solving the puzzle, as data on these controversial points as well as on order of dissociation of the two ligands are incomplete [17].

We here perform extended bias exchange metadynamics simulations (BE-MTD) [18]–[20] aimed at establishing the reciprocal influence of the Na^+^ and Gal in their dissociation mechanism and at characterizing the kinetics and thermodynamics of the process. Our study shows that (i) the Na^+^/Gal interplay along the dissociation path is minimal and it is limited only to the initial displacement of both Na^+^ and Gal from their binding sites; (ii) the dissociation of both Na^+^ and Gal occurs with free energy barriers of about 11–12 kcal/mol, and the rate limiting step is associated to conformations in which Na^+^ and Gal are more than 10 Å far apart from their binding sites; (iii) no gating role can be assigned to Y263. Simulation of the Y263F mutant reveals a rather significant change in the binding site of Gal, confirming that this residue has an important functional role, even if it does not act as a gate.

## Materials and Methods

### System Setup

The setup is the same we used in our previous work [15]. The model of vSGLT was built using the chain A of the 3 Å resolution crystal structure (PDB accession code 3DH4 [6]). The first helix, partially solved, was reconstructed from a more recent crystal structure (PDB code 2XQ2 [14], 2.7 Å resolution). The missing atoms of side chains of residues K124, V185, R273, K454, K547 were built using SwissPDBViewer [21] application. Residues S31 to L46, located between transmembrane helix (TM) 1 and TM2, and residues Y179 to A184, between TM5 and TM6, were built using Loopy [22] program. We here number the helices like in Ref [6]. The final monomeric structure contained 539 residues (S9 to K547). The protein was embedded in a pure, pre-equilibrated 1-palmitoyl-2-oleilphosphatidylcholine (POPC) lipid model (kindly supplied by T. A. Martinek) [23], [24] using the gmembed [25] tool of GROMACS4 [26] and then it was oriented following OPM [27] database model. Afterward the system was neutralized and solvated with TIP3P [28] water molecules (80969 total atoms in a box size of 97.6×96.7×85.1 Å^3^). Simulations were carried out with GROMACS4 [26] package using Amber03 [29] force field for protein, GAFF [30] for galactose and for membrane the parameters supplied by T. A. Martinek [23]. For more details, see Supplementary Information (S1 Text).

### Bias-Exchange Metadynamics Simulations

The starting point of this study was the structure of vSGLT in an ion-retaining state obtained from our previous MTD simulations [15]. In order to study the binding/dissociation of galactose and its coupling with the binding/dissociation of sodium ion by using BE-MTD, we exploited nine different collective variables using the Plumed plugin [31]. The novelty of this technique with respect to standard MTD is that a large number of collective variables can be employed at the same time, allowing thus the study of complex (bio)chemical processes, as in this case the simultaneous dissociation of both Na^+^ and Gal. Seven of the CVs were reserved to the Gal. In particular, to assess the controversial role of Y263 we used 1) a combination of two dihedral angles of Y263 (C-C*α*-C*β*-C 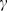 and C*α*-C*β*-C 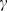 -Cδ) using the alpha-beta similarity keyword of Plumed [31] and 2) the hydrogen bonds (H-bonds) between Y263 and N64. To focus on the dissociation of Gal from its binding site we used: 3) the distance of galactose from its binding site (represented by the center of mass (COM) of selected residues, see S1 Table); 4) the H-bonds between galactose and its binding site; 5) the H-bonds between galactose and the likely exiting pathway [13], [14], [16]; 6) the radius of gyration of a group of atoms belonging to the galactose binding site; 7) a path collective variable, which describes the progression of the galactose along its exit channel [32]. This variable, introduced in the simulation after a spontaneous exit of the galactose was observed biasing the Gal dissociation process with the other variables, is defined by a set of 7 reference conformations. Two additional variables were used to characterize the release mechanism of Na^+^: 8) the distance between the sodium ion and its binding site (defined by the COM of selected residues, see S1 Table) and 9) the coordination number between sodium and four residues of the binding site (carbonyl oxygens of I65 and A361 and hydroxyl oxygens of S364 and S365).

Initially, each walker was biased by only one CV. After 740 ns, in order to improve the statistics and allowing a faster convergence of the free energy profile (see S2 Text) more walkers were added biasing the same CVs. In this way, we simulated a total of 1400 ns using at most 16 walkers. Parameters like gaussians width (ds), intervals, walls were added/changed and adapted for a better and faster convergence of the simulation (see S2 Text, S1 Table). Simulations of ligands dissociations were also performed in the absence of Na^+^ in order to assess the role of the ion. Moreover, we have performed an additional simulation starting from the Y263F mutant to clarify the role of this residue in shaping the free energy landscape. At this scope we elongated the BE-MTD simulation mutating Y263F (for a total of 15 ns*16 CVs  = 240 ns), maintaining all the CVs and their parameters used in the wild type (WT) simulation (S3 Text).

### Analysis

All structural and free energy analyses were performed using METAGUI [33], a VMD [34] interface for analyzing metadynamics and molecular dynamics simulations.

The error of the free energy profiles was calculated as the standard deviation of two different time averages of the biased potential in the first and the second part of the converged interval of the simulation.

In order to assess the role of selected residues along the ligands dissociation paths we calculated the average interaction energies at the relevant minima and transition states. We considered the van der Waals and coulombic interactions. We remark that this analysis is qualitative and is meant only to provide a picture of the role of selected residues in the relative stabilization/destabilization of transition states and minima, as shown in other studies [35], [36].

H-bond analysis was performed using Plumed [31].

## Results

### Galactose Exit Pathway

**Figure 1:**
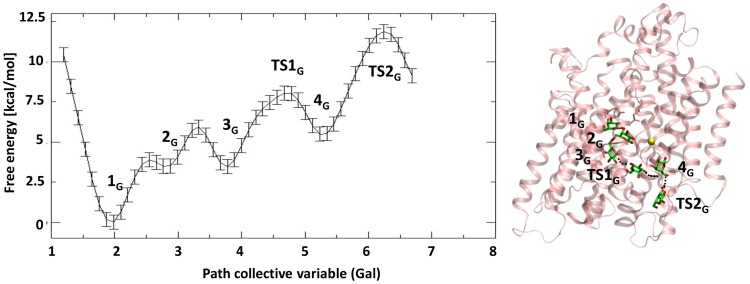
Exit pathway of galactose. On the left is shown the free energy profile (kcal/mol) along the path collective variable representing Gal exit. On the right it is reported the position of the Gal in the different minima along exit pathway on the top of the protein. This latter is depicted in pink cartoons, the substrate in licorice, while the Na^+^ is depicted as a yellow sphere. The curved path is connected by dark dashed lines.

We first investigated the dissociation path of Gal. The projection of the free energy along the path collective variable, representing the progression of Gal along its exit channel, is reported in Fig. 1. For sake of clarity, we add a subscript *_G_* for the states relative to the Gal exit path, and a subscript *_Na_* for those relative to the ion path. Initially, Gal is in the deepest minimum (Min 1*_G_*), which is the same binding site identified in the crystal structure (the RMSD of the heavy atoms of residues within 4 Å of Gal with respect to the conformation in the crystal structure is 1.0 Å (±0.1)). The substrate is stabilized by an extended H-bond network with E88, Q428, Q69, E68, N64 (S2 Table). Residue Y263 (OH) interacts with N64 (HN 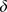). There are 2-3 water molecules in the binding site, interacting with the substrate. While Gal is in its binding site, Na^+^ is coordinated by three water molecules and three residues (A62, I65 and S365). This corresponds to the ion-retaining binding site of the inward-facing conformation of vSGLT identified in our previous work [15]. In analogy with our previous study we name this site LC1.

The next free energy minimum along the exit path of the substrate is Min 2*_G_*, where, consistently with the suggestion of Li et al. [16], Gal undergoes a lateral displacement toward a position in which it is only partially shielded by the ring of the Y263. From this point, the substrate will find its way out by rotating about 90 degrees (assuming a conformation in which its ring is roughly parallel to the protein axis) and continues his progression along a curved path beyond Y263. This residue is at the edge of the hydrophilic cavity and the presence of water molecules confers flexibility to it, which hence is able to accommodate to the passage of Gal (S1 Fig.). In this new position, several water molecules enter the binding site, while the substrate (C6-O) interacts with T431 (HO 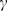) and, through a water bridge, with N142. In this minimum residue E68 assumes a new rotameric conformation. Indeed, its side chain, initially heading toward the Gal binding site, rotates toward the Na^+^ binding site, making one or two H-bonds with S66, a conserved residue across the SSS family (E68 (O 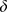 1) with S66 (HN) and E68 (O 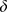 2) with S66 (HO 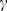)). The interactions of N64 (O) with galactose (HO-C2) and N64 (HN 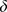) with Y263 (OH) are still present, even if characterized by large fluctuations. A qualitative analysis of the interaction energies between the substrate and selected residues shows that in the first two minima the van der Waals interactions regard the substrate and Y263, while the electrostatic interactions involve Gal-N64 (S3 Table).

Afterwards, the substrate, hydrated by 4–5 water molecules, enters into a narrow cavity created by the residues N267, Q268, W134, T431, V434, transiently interacting with N142 and Y262 (Min 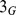). The H-bonds of E68 (O 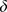 1) with S66 (HN) and E68 (O 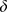 2) with S66 (HO 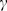) contribute to stabilize Gal in this minimum (S2 and S3 Tables). Y263 and N64 become very flexible as they can not form reciprocal H-bonds.

After overcoming a transition state (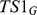), where the substrate is partially hydrated, Gal finds another minimum (Min 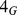). Here, it is almost fully hydrated, and surrounded by S368, V185, and the TM2-I, TM9, TM6, above loop TM5-6 and it is right below the sodium binding site, inside the hydrophilic cavity of the transporter. Gal (C1-OH and C2-OH) makes H-bonds with D189 (O 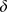) and Gal (C6-OH) with A184 (carbonyl oxygen). Residue D189 is highly conserved throughout the SSS family and it has been experimentally seen to play an important role for a correct Na^+^-Gal cotransport and cation selectivity [37], [38]. We see here that it is also involved in the exit path of galactose, contributing to the stabilization of this minimum. Here, the aromatic ring of Y263 maintains an orientation similar to the crystal structure, while the sidechain of N64 assumes a new conformation, pointing toward the carboxylic group of E68. The H-bond between E68-S66 is present also when Gal is in this minimum (S2 Table).

In order to leave this site, moving deeper in the hydrophilic cavity, Gal has to overcome a transition state (TS2G) with ΔF# of 11.9±0.4 kcal/mol with respect to the minimum, which corresponds to the largest free energy barrier of the exit pathway. The breaking of the H-bonds between Gal and D189 contributes to the barrier, as suggested by the interaction energies among Gal and D189 along the path (S2 and S3 Tables).

At 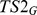, the substrate is at the protein surface and, although being hydrated, it is still interacting with a few surface residues forming a H-bond (Gal (C3-OH) with N371 (O 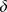) and hydrophobic interactions with other residues (G181, L182 (on loop TM5-6), V396 (TM10), N371 and T375 (TM9) (S2 and S3 Tables).

Remarkably, the free energy barriers associated to the exit path of Gal are significantly higher than those calculated by Watanabe et al. [14]. This is most probably due to the fact that our simulations start from a stable ion-retaining state of the transporter, and since a subtle cooperativity between Na^+^ and Gal is observed at the very beginning of the path, MD runs starting from the crystal structure, as those of Watanabe et al. [14], which corresponds to an ion-releasing state, may lead to simulate a different dissociation process.

Since Y263F mutation has been observed to impair the transport mechanism, to further check the controversial role of Y263 in the dissociation of the substrate, we performed a BE-MTD simulation of the mutant using the same setup of the WT simulation. Looking at S2 Fig., we can clearly see a different profile, where the second minimum becomes the global one, more stable and broader than the first minimum. In short, Min1-Y263F corresponds to Min1-WT, while Min2-Y263F is broad and thus characterized by different configurations of Gal (containing among them Min2-WT minimum). Their relative free energy has changed, meaning that Y263 decides in this transporter the relative stability of the minima characterizing the releasing path of the substrate. Thus, this mutation has a role in reshaping the free energy surface of Gal exit path. The stabilization of the other minimum does not seem to influence the barrier height significantly, but it may hamper Gal from assuming a position necessary to undergo the inward-outward conformational change, affecting in turn the overall transport cycle, in line with experimental findings [14]. We remark that the change in the free energy profile of the releasing of Gal caused by this mutation does not imply a gating role for this residue.

### Sodium Ion Exit Pathway

**Figure 2:**
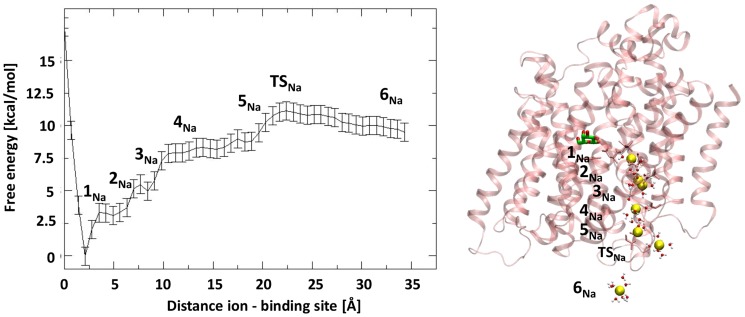
Exit pathway of sodium ion. Free energy profile (kcal/mol) projected along the distance ion - center of mass of its binding site (Å) is displayed. On the right the position of the different states along the exit path of Na^+^ are shown on the top of the protein.

The dissociation path of Na^+^ is characterized by an overall free energy barrier of 11.1 (±0.7) kcal/mol and by the presence of a few faint metastable states (see Fig. 2). The most stable ion binding site is LC1 in which the ion is coordinated by three water molecules and three residues (A62, I65 and S365). As soon as the ion starts moving toward the cytoplasm, it loses its coordination with I65; then, it approaches the mouth of the binding site keeping the interaction with A62 and coordinating S364 and D189. This latter is often found to bind Na^+^ along the exit. This state was called PC in our previous work [15].

In this configuration the side chain of E68 rotates from a configuration in which it heads toward the Gal binding site to a new conformation in which it forms hydrogen bonds with S66. In State 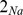 the ion, at almost 5 Å from its binding site, has overcome D189, moving deeper into the hydrophilic cavity, and it is fully hydrated. Then, it continues descending into the cavity coordinating G181 (loop 5–6) and S368 (on TM9) and four water molecules (State 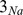). Remarkably, D189 interacts with the ion through water bridges, accompanying it from LC1 to State 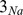, confirming its important role in the exit pathway of 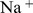 [15], [37], [38]. After interacting with L182 (loop 5-6) (State 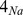), it reaches State 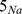, which is at approximately 1.8 Å from the binding site. Here, Na^+^ is coordinated by a few residues of loop 1–2 and by 3–4 water molecules. It finally overcomes 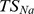, where it is still transiently coordinated by D43, R400, and even by a POPC molecule. Thus, the total free energy barrier is due to cumulative energy cost of small structural changes accompanying the Na^+^ release without the formation of any stable intermediate. After the 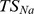 Na^+^ is quite delocalized, in a vestibule mainly formed by loops TM9–10, TM1–2 and loop TM5–6. It is important to note that the two ligands, starting from different binding sites, exit the protein through the same hydrophilic cavity (characterizing the inward-facing conformation) communicating to the cytoplasm.

### Is the Release Mechanism of the Galactose and the Sodium Ion Cooperative?

**Figure 3:**
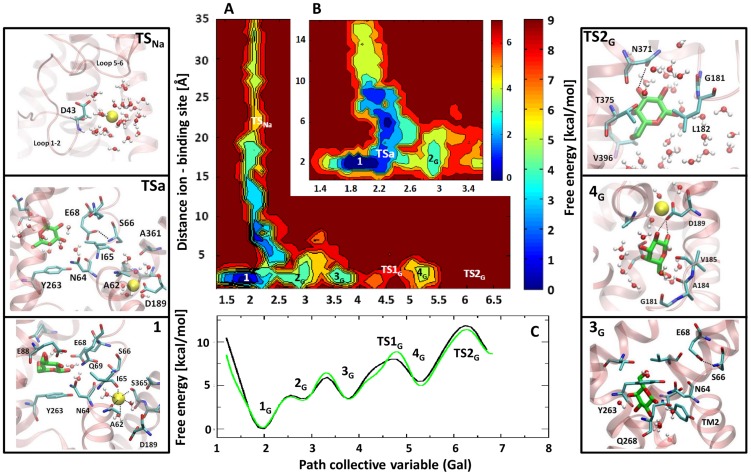
The cooperativity in the release mechanism. (A) Free energy surface (kcal/mol) depicted with respect to the distance between Na^+^ and its binding site (Å), and the galactose exit path. Note that the deepest minima for both exit pathways are the same, i.e. 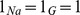. (B) Close view of the initial part of the FES. (C) The projection of the free energy (kcal/mol) along the path collective variable representing Gal exit. Black and green lines correspond to the simulations carried out in presence or in absence of Na^+^. On the two sides of the image, the most relevant structures corresponding to minima and transitions states (TS) are depicted.

In order to investigate possible cooperative effects in the release mechanism of Na^+^ and Gal, in Fig. 3 we report a projection of the free energy surface (FES) as a function of two CVs, the distance between the ion and its site and the path variable of Gal. It is possible to note that the deepest minimum for both exit pathways is the same, i.e. 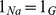. Thus, it is labeled as Min 1. A zoom of the free energy landscape in the region close to the binding sites is also reported. The shape of the free energy landscape clearly suggests an interplay between the two ligands. Indeed, upon displacement of Gal from its binding site to move toward Min 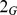, the Na^+^ loses its coordination with I65 and moves toward a site with a reduced number of coordinating residues, the PC site (state 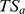). The residue linking the two binding sites is the E68. Indeed, upon Gal displacement from Min 1, E68, initially heading toward the Gal binding site, rotates toward the Na^+^ binding site, establishing one H-bond with S66 (HN) (S3 Table), a conserved residue across the SSS family. This functional rotation of E68 is also observed between the holo (PDB 3DH4) and the apo (PDB 2XQ2) forms of the vSGLT crystals. These results are in line with the previous hypothesis suggesting that the departure of Na^+^ from its stable putative ion-retaining site, LC1, toward the PC site triggers the conformational changes at the basis of Gal displacement from the binding site, heading to the second metastable minimum of the path (Min 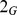). However, the free energy barrier associated to this initial displacement is very small and the highest barriers lay further along the dissociation path of Na^+^ and Gal. This fact, along with the overall L-shape of the free energy for large values of the collective variables (see Fig. 3), indicates that the rate limiting steps of the release of the ion and the substrate are independent. Indeed, the values of the two CVs (the path collective variable of Gal and the distance Na^+^ - binding site) at the highest transition state (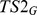) of Gal exit are 6.2 and 3 Å. While, those at the 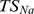 of Na^+^ exit are 2.2 and 21 Å. Namely, at the transition state of Gal, Na^+^ is close to its binding site, and viceversa. In order to quantitatively verify this point, we computed the free energy of Gal exit in absence of Na^+^. The free energy profile of Gal dissociation in the absence of Na^+^ is practically identical to the profile in the presence of Na^+^, confirming unambiguously this important result (Fig. 3, panel C).

## Discussion

In this work we used BE-MTD to study the binding/dissociation mechanism of the two ligands of vSGLT symporter. We observed that the minimum free energy exit pathway of the galactose does not involve any rotameric transition of the side-chain of Y263. Indeed, as already observed [16], Gal circumnavigates the so-called inner gate Y263 and proceeds along the hydrophilic cavity. However, our simulation of the mutant points to a possible functional role of Y263 in determining the relative stability of the minima observed along the Gal exit path. The global free energy minimum for the mutant and for the WT turns out to be different.

The main barriers characterizing the releasing mechanism are of the order of 11–12 kcal/mol for both the ion and the substrate. These barriers are significantly higher than those reported by Watanabe et al. [14]. This is possibly due to the fact that the free energy space explored in our case includes an occluded state of the transporter, with both ligands stable in their binding sites, while the work of Watanabe et al. [14], starting from a different structure, may simulate a different process, the departure of the ion from an ion-releasing state. As also mentioned in our previous work, our starting structure is only a possible candidate of an ion retaining state. However, this binding site of Na^+^ is the same identified in our previous work [15], by independent metadynamic simulations, demonstrating the reliability of our results. This ion-retaining site is also consistent with the observation of Faham et al. [6] pointing to an important role of S365 for the Na^+^-dependent transport of Gal. Moreover, Loo et al. [39] observed that a mutation of residue S392 of Na2 site of hSGLT1 (that works with stoichiometry 2 Na^+^: 1 sugar) affects the binding of both sugar and the second ion. In this case, a straightforward comparison with the corresponding S364 of vSGLT is not possible due to the different number of ions needed to activate Gal transport (stoichiometry 1 Na^+^: 1 sugar).

Concerning the reliability of the mechanism we propose, we remark that our barriers are affected by errors of the order of 0.7 kcal/mol due to the convergence of our BE-MTD simulations. Moreover, like in all the simulations based on classical molecular dynamics, our results could be affected by systematic errors due to the force field. These errors can alter the value of the barriers, but are unlikely to affect our main finding. Namely the fact that Na^+^ and Gal release are independent. Finally, we remark that we have not attempted computing the binding free energy ΔF of the two ligands. The value of the free energy at the maximum distance from the binding site is not a quantitative estimate of the ΔF, due to residual interactions of the ligands with the surface of the protein in the final states of our free energy profiles. The aim of our work was to reveal the interplay of Na^+^ and Gal during their dissociation mechanism focusing on the free energy barriers rather than on the binding free energies.

As we already underlined, the barriers calculated in this study are large, possibly of the same order of magnitude of those associated with the inward-outward conformational switch. Indeed, the transition rate associated with the crossing of our barriers is approximately 1 s^−1^ (assuming prefactor of 10^−8^ s^−1^). This value is in line with kinetic experimental data. Indeed, the galactose turnover in vSGLT was estimated to be around 0.4 s^−1^ by Turk et al. [40], or on the order of tens of ms in other works [11], [41]–[43]. Lapointe and coworkers measured the turnover rate of hSGLT1, finding values of 8 s^−1^ or, being near V*_max_* conditions, 13 s^−1^. Moreover, a transition rate of 50–60 s^−1^ was found for hSGLT1 [17], [42]. These values correspond to a free energy barrier in the range of 11–15 kcal/mol, consistently with our results.

Importantly, our simulations provide for the first time direct insights on the possible cooperativity between Na^+^ and Gal for their release mechanism toward the cytoplasm. A small interdependence is observed only at the very beginning of the ligands release process, with residue E68 playing a central role in the communication between the two binding sites. Remarkably, this intercommunication occurs far from the point of the free energy profile associated to the highest free energy barriers. Our simulations, carried out in the absence of Na^+^, reveal that the whole free energy profile of Gal exit is essentially unaffected, Fig. 3 (green line, panel C). The lack of a marked cooperativity in the release mechanism of Gal and Na^+^ from the binding site is at first sight surprising. However, it is likely that the cooperativity might be associated to the first steps of the transport cycle, when the symporter, in the outward-facing conformation, binds the sodium ion and then the substrate, and their binding triggers the outward-to-inward facing conformational change, as observed in the LeuT-fold superfamily [8], [44]–[47].

**Figure 4:**
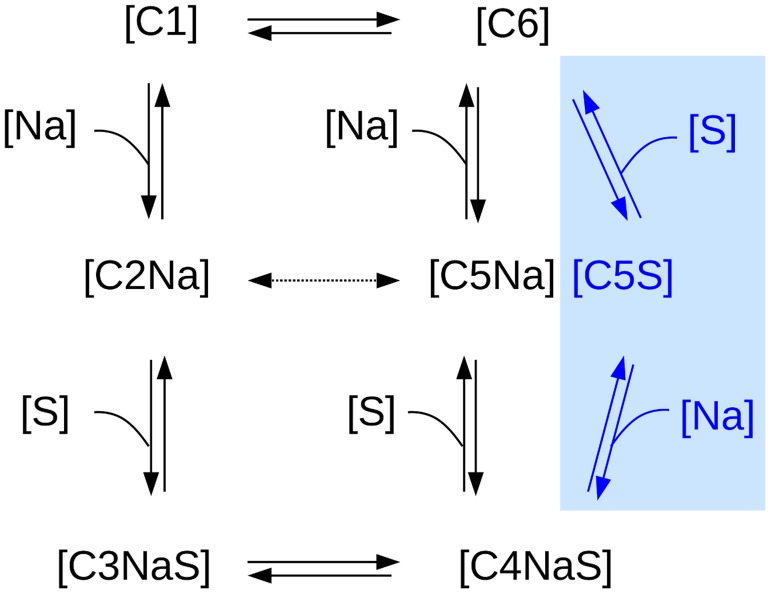
Six-state kinetic model for Na^+^-galactose cotransporter updated to a branched six-state model. In the absence of ligands, the transporter can be in two states: outward or inward-facing conformation (C1 and C6, respectively). After the binding of Na^+^ to the outward conformation (C2Na), the substrate enters the protein and finds its site (C3NaS). This step is then followed by the crucial event that sees the transporter switching to the inward-facing conformation (C4NaS). In case the transport follows the dashed line, namely passing from C2Na to C5Na, a uniport of Na^+^ ion happens. From the ligands-loaded inward conformation (C4NaS) the protein can lose at first the substrate (C5Na), as suggested in Ref. [11], [48], or, based on our results (blue region), in an independent way characterized by similar barriers, the ion (C5S).

Due to this non-cooperativity in the Gal and Na^+^ release mechanism from the inward-facing conformation of vSGLT and to the almost identical rate limiting free energy barriers, we propose to extend the six-state kinetic model introduced by Wright and coworkers [11], [48] by adding one more state, Fig. 4 (blue region). Indeed, we suggest that, from the ligand bound inward-facing conformation, the transporter can follow independently two paths for Gal and Na^+^ release. The very similar free energy barriers observed for the Na+ and Gal dissociation from the inward-facing conformation may be in part responsible for the difficulties encountered experimentally in providing a detailed and clear picture for this part of the transport path of hSGLT [11], [17], [48].

We also observe that the crystal structure of the inward-facing conformation in the apo form of (PDB code 2XQ2) [14] differs in the presence of a kink of a few degrees in TM2-I (intracellular half) and the side chain of E68 heading toward S66. Remarkably, these structural features are observed during the Gal release pathway, suggesting that starting from a Na^+^ occluded and holo state of vSGLT (PDB code 3DH4) [6], we are able to visit these structural features of the apo state captured crystallographically (PDB code 2XQ2) [14].

An important process associated to this transporter is the permeation of water, whose precise mechanism is still under debate [49], [50]. The two mechanisms considered more viable are the active cotransport [49], [51], where water flux is coupled to ion/solute flux, or the passive permeation [52], where the accumulation of the solutes near the intracellular side of the membrane during solute transport induces a flux of water as a response to the local osmotic gradient. A detailed analysis of this controversial issue is beyond the scope of our study. However, in line with the passive mechanism [53], [54], we observe that: (i) the water molecules permeate easily through the whole protein during the releasing process; (ii) the water is free to enter from the cytoplasm into the hydrophilic cavity and then into the binding sites. Consistently with Ref. [45], [55], in our simulations water molecules help the breaking of the H-bonds that keep the ligands bound to the protein, playing an important role in the whole releasing pathway. Their presence at the edge of the Gal binding site confers, indeed, flexibility to Y263, facilitating the initial displacement of the substrate (S1 Fig.).

## Supporting Information

S1 Figure **Flexibility of Y263.** Min 1 (purple), Min 2 (green) and Min 3 (blue) of the Gal exit path are shown. Residues Y263, N64 and Gal are reported in licorice. In order to capture the flexibility of the Y263, several conformations of the same minimum are represented. Water molecules, freely coming from the hydrophilic cavity, are shown in red dotted spheres.
(TIF)

S2 Figure **Free energy profile of wild type and the mutant.** The free energy profile of wild type (WT) system along the path collective variable of Gal (black line) and that of the mutant Y263F (green line).
(TIF)

S1 Table **CVs used in this study.** The units of the CV3, CV6 and CV8 are expressed in nanometers, CV1 in radiants. The galactose binding site was defined by the C*α* of residues Q69, N260, K294 and Q428. The ion binding site was defined by the C*α* of A62, I65, S66, V363, S364 and S365.
(PDF)

S2 Table **The average distances (Å), the standard deviation and the life time (%) of relevant H-bonds along the Gal dissociation path.**
(PDF)

S3 Table **Average electrostatic and van der Waals interaction energies (kcal/mol) between galactose and residues Y263, N64 and D189, at selected minima and transition states visited along the exit path of Gal.**
(PDF)

S1 Text **Molecular dynamics protocol.**
(PDF)

S2 Text **Parameters for BE-MTD.**
(PDF)

S3 Text **Mutant.**
(PDF)
