# Supplementary figures and images for: Metadynamics Simulations Reveal a Na+ Independent Exiting Path of Galactose for the Inward-Facing Conformation of vSGLT
Source: PLoS Comput Biol. 2014 Dec 18;10(12):e1004017. doi: 10.1371/journal.pcbi.1004017 (PMC4270436; doi:10.1371/journal.pcbi.1004017)

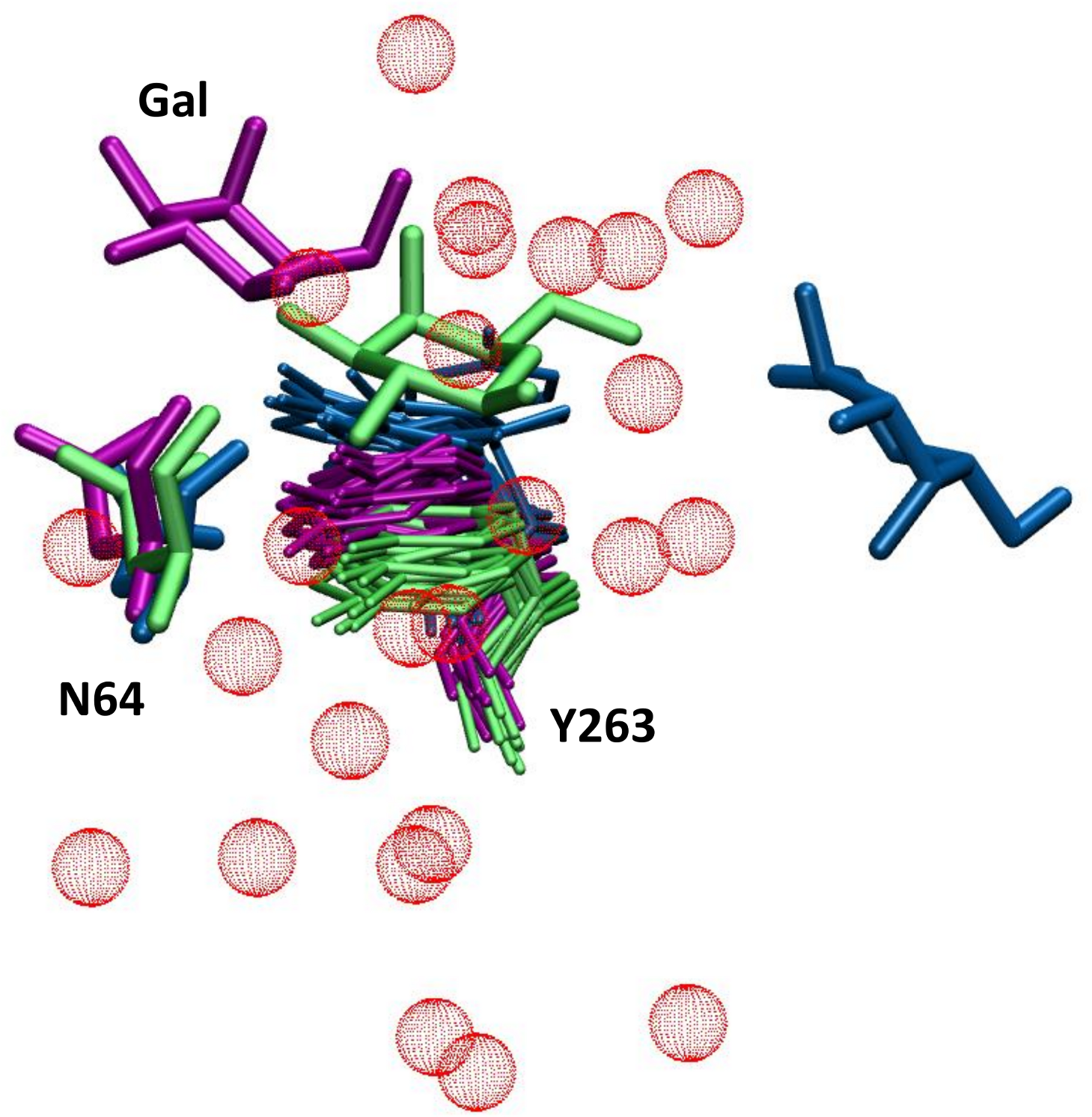

Supplement: S1 Figure — Flexibility of Y263. Min 1 (purple), Min 2 (green) and Min 3 (blue) of the Gal exit path are shown. Residues Y263, N64 and Gal are reported in licorice. In order to capture the flexibility of the Y263, several conformations of the same minimum are represented. Water molecules, freely coming from the hydrophilic cavity, are shown in red dotted spheres. (TIF) [file pcbi.1004017.s001.tif]

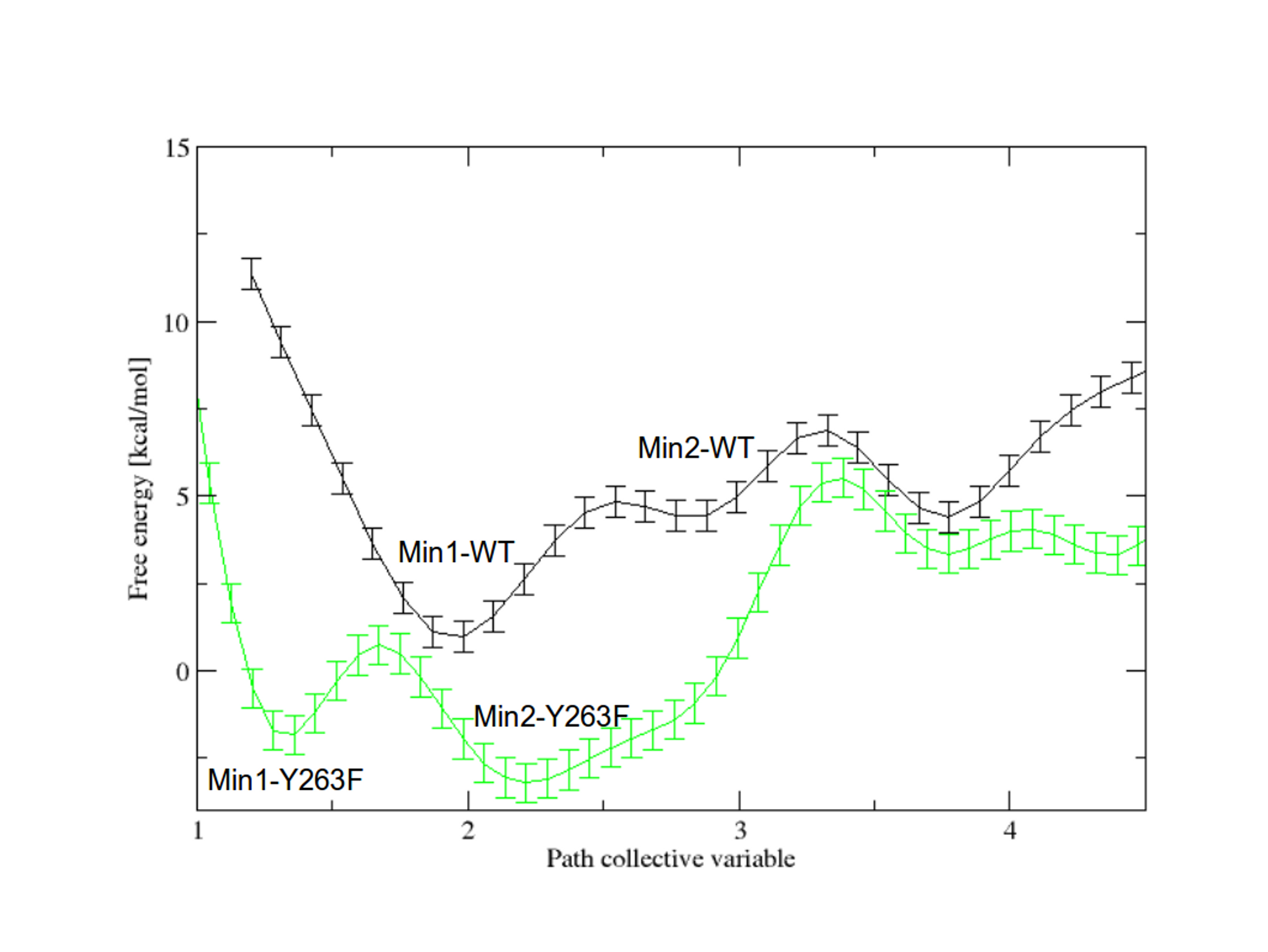

Supplement: S2 Figure — Free energy profile of wild type and the mutant. The free energy profile of wild type (WT) system along the path collective variable of Gal (black line) and that of the mutant Y263F (green line). (TIF) [file pcbi.1004017.s002.tif]
