# Supplementary material for: Metadynamics Simulations Reveal a Na+ Independent Exiting Path of Galactose for the Inward-Facing Conformation of vSGLT
Source: PLoS Comput Biol. 2014 Dec 18;10(12):e1004017. doi: 10.1371/journal.pcbi.1004017 (PMC4270436; doi:10.1371/journal.pcbi.1004017)
Supplement: S1 Table — CVs used in this study. The units of the CV3, CV6 and CV8 are expressed in nanometers, CV1 in radiants. The galactose binding site was defined by the Cα of residues Q69, N260, K294 and Q428. The ion binding site was defined by the Cα of A62, I65, S66, V363, S364 and S365. (PDF) [file pcbi.1004017.s003.pdf]

Table S1. CVs used in this study.

CV	δs	Upper Wall	Interval
1. Dihedral angle Y263	0.025	-	0.1 - 1.9
2. H-Bonds Y263-N64	0.35	-	0.1 - 2
3. Distance Gal-site	0.05	3.5	0 - 3.5
4. H-Bonds Gal-site	1	-	1 - 30
5. H-Bonds Gal-path	1	-	1 - 31
6. Radius of Gyration Gal site	0.02	1.05	0 - 1.05
7. PCV	0.3	-	1.2 - 6.8
8. Distance Na^+-site	0.05	3.5	0 - 3.5
9. Coordination Na^+ site	0.1	-	0.1 -3.5

The units of the CV3, CV6 and CV8 are expressed in nanometers, CV1 in radiants. The galactose binding site was defined by the Cα of residues Q69, N260, K294 and Q428. The ion binding site was defined by the Cα of A62, I65, S66, V363, S364 and S365.
