# Supplementary material for: Metadynamics Simulations Reveal a Na+ Independent Exiting Path of Galactose for the Inward-Facing Conformation of vSGLT
Source: PLoS Comput Biol. 2014 Dec 18;10(12):e1004017. doi: 10.1371/journal.pcbi.1004017 (PMC4270436; doi:10.1371/journal.pcbi.1004017)
Supplement: S2 Table — The average distances (Å), the standard deviation and the life time (%) of relevant H-bonds along the Gal dissociation path. (PDF) [file pcbi.1004017.s004.pdf]

Table S2. The average distances (Å), the standard deviation and the life time (%) of relevant H-bonds along the Gal dissociation path.

HB	Mean (st.dev.)	% ≤ 3 Å	% ≤ 3.2 Å
Min 1_G			
E68OE1-GalO1	2.70 (± 0.27)	94.6	95.0
E88OE2-GlaO3	2.62 (± 0.095	100.0	100.0
Q428NE2-GalO6	3.88 (± 1.14)	22.7	44.1
N64ND2-GalO2	3.28 (± 0.27)	13.2	45.5
N64ND2-Y263OH	3.09 (± 0.21)	38.2	75.0
Q69NE2-GalO2	3.03 (± 0.15)	50.0	86.4
Min 2_G			
GalO6-T431OG	2.92 (± 0.26)	72.9	90.6
GalO2-N64O	2.92 (± 0.21)	68.8	92.7
S66N-E68OE2	2.78 (± 0.07)	100.0	100.0
S66OG-E68OE1	2.76 (± 0.33)	88.5	89.6
N64ND2-Y263OH	3.17 (± 0.26)	28.1	64.6
Min 3_G			
GalO1-T431OG	3.40 (± 1.17)	50.6	74.7
S66N-E68OE1	3.10 (± 0.20)	35.6	71.3
S66N-E68OE2	2.81 (± 0.12)	95.4	97.7
S66OG-E68OE1	2.80 (± 0.42)	87.4	87.4
GalO2-N142OD	4.42 (± 1.94)	44.8	47.1
GalO3-N142OD	3.84 (± 1.41)	57.5	58.6
GalO5-Y262OH	3.62 (± 1.32)	36.8	54.0
Min 4_G			
GalO1-D189OD2	2.76 (± 0.78)	90.4	97.9
GalO2-D189OD2	2.77 (± 0.58)	94.9	98.6
GalO4-S368OG	2.98 (± 0.55)	68.9	90.4
GalO6-A184O	3.06 (± 0.94)	74.4	87.7
$TS2_G$			
GalO3-N371OD	3.54 (± 1.09)	52.1	64.6
