# Supplementary material for: Metadynamics Simulations Reveal a Na+ Independent Exiting Path of Galactose for the Inward-Facing Conformation of vSGLT
Source: PLoS Comput Biol. 2014 Dec 18;10(12):e1004017. doi: 10.1371/journal.pcbi.1004017 (PMC4270436; doi:10.1371/journal.pcbi.1004017)
Supplement: S3 Table — Average electrostatic and van der Waals interaction energies (kcal/mol) between galactose and residues Y263, N64 and D189, at selected minima and transition states visited along the exit path of Gal. (PDF) [file pcbi.1004017.s005.pdf]

Table S3. Average electrostatic and van der Waals interaction energies (kcal/mol) between galactose and residues Y263, N64 and D189, at selected minima and transition states visited along the exit path of Gal.

State	Coul Y263	LJ Y263	Coul N64	LJ N64	Coul D189	LJ D189
1_G	0.10 (± 0.45)	-4.04 (± 0.65)	-2.90 (± 1.39)	-1.40 (± 0.70)	0	0
2_G	-0.40 (± 0.52)	-4.38 (± 0.66)	-3.36 (± 1.75)	-1.68 (± 0.80)	0	0
3_G	0.05 (± 0.15)	-0.61 (± 0.41)	0	0	0	0
$TS1_G$	0.01 (± 0.02)	-0.10 (± 0.03)	-0.01 (± 0.04)	-0.15 (± 0.06)	-0.02 (± 0.03)	-0.02(± 0.02)
4_G	0	0	-0.03 (± 0.03)	-0.20 (± 0.05)	-22.82 (± 6.38)	2.39 (± 2.22)
$TS2_G$	0	0	0	0	0	0
