# Supplementary material for: Metadynamics Simulations Reveal a Na+ Independent Exiting Path of Galactose for the Inward-Facing Conformation of vSGLT
Source: PLoS Comput Biol. 2014 Dec 18;10(12):e1004017. doi: 10.1371/journal.pcbi.1004017 (PMC4270436; doi:10.1371/journal.pcbi.1004017)
Supplement: S1 Text — Molecular dynamics protocol. (PDF) [file pcbi.1004017.s006.pdf]

Molecular Dynamics Protocol

A computational protocol similar to our previous work was used [15]. For the ions, we used the parameters of Amber force field ff03 (corresponding to the library of 1994). Namely, Na+ σ = 3.32840e−01 and = 1.17152e−02, Cl− σ = 4.40104e−01 and = 4.18400e−01. [29] For the galactose, RESP [56] charges were calculated fitting an electrostatic potential calculated using the 6-31G* basis set and B3LYP [57, 58] functional and the Gaussian03 [59] program. The simulations were performed in periodic boundary conditions at 310 K using the Nose-Hoover thermostat [60] and Parrinello-Rahman barostat [61, 62] with a semisotropic pressure coupling type and a time step of 2 fs.

The BE-META calculations were conducted in the NVT ensemble.

During all the simulations, we took care in particular that important parameters of the membrane, such as the Area Per Lipid (APL) and thickness, were in agreement with experimental values [63].

References

56. Bayly C, Cieplak P, Cornell W, Kollman P (1993) A well-behaved electrostatic potential based method using charge restraints for deriving atomic charges: the resp model. J Phys Chem 97: 10269–10280.

57. Lee C, Yang W, Parr R (1988) Development of the colle-salvetti correlation-energy formula into a functional of the electron density. Phys Rev B 37: 785.

58. Stephens P, Devlin F, Chabalowski C, Frisch M (1994) Ab initio calculation of vibrational absorption and circular dichroism spectra using density functional force fields. J Phys Chem 98: 11623–11627.

59. Frisch M, Trucks G, Schlegel H, Scuseria G, Robb M, et al. (2004) Gaussian 03, rev. c. 02. gaussian. Inc, Wallingford, CT .

60. Evans D, Holian B (1985) The nose–hoover thermostat. J Chem Phys 83: 4069.

61. Parrinello M, Rahman A (1981) Polymorphic transitions in single crystals: A new molecular dynamics method. J Appl Phys 52: 7182–7190.

62. Nose S, Klein M (1983) Constant pressure molecular dynamics for molecular systems. Mol Phys 50: 1055–1076.

63. Kučerka N, Tristram-Nagle S, Nagle J (2006) Structure of fully hydrated fluid phase lipid bilayers with monounsaturated chains. J Membrane Biol 208: 193–202.
