# Supplementary material for: Metadynamics Simulations Reveal a Na+ Independent Exiting Path of Galactose for the Inward-Facing Conformation of vSGLT
Source: PLoS Comput Biol. 2014 Dec 18;10(12):e1004017. doi: 10.1371/journal.pcbi.1004017 (PMC4270436; doi:10.1371/journal.pcbi.1004017)
Supplement: S2 Text — Parameters for BE-MTD. (PDF) [file pcbi.1004017.s007.pdf]

Parameters for BE-MTD

In the Table S1 the values of the width (δs) of gaussians are reported. Moreover, some parabolic restraints (walls) (force constant, K, 5000 kJ mol−1 nm−2) were introduced, in order to avoid the sampling of regions which were not relevant for the ligand dissociation process. The positions of these walls are reported as well. In order to reduce the error close to the boundaries we applied the approach in Ref [64], namely we set the force equal to zero beyond an interval, specific for each CV. The gaussians were added every 5 ps with a height (W) equal to 0.08 kJ/mol for all the CVs. The exchanges between the bias potentials of the different variables were periodically attempted every 20 ps. We started the simulation with 8 walkers using all CVs except the path collective variable. After 740 ns we added other 4 walkers for the distance between Gal and the center of mass of its site, at 820 ns we introduced 4 more walkers biased on the path collective variable of the Gal.
Reconstruction of the 2D-FES. Once the bias potential does not change in shape, it is possible to construct the free energy of the system. The CV space is subdivided so that all the frames of BE-MTD trajectories are grouped in microstates whose members are close to each other in the CV space [19, 20]. The free energy of each microstate is then estimated by a weighted-histogram analysis (WHAM) approach [65], where the effect of the bias is removed. It is important to highlight that when reconstructing the free energy landscape it is not necessary to use all the CVs explicitly biased, as some of them might prove a posteriori to be less relevant for the process under investigation or to be strongly correlated with other variables. Indeed here, despite biasing 9 different CVs, we used three CVs, the two distances and the path collective variable.

References

64. Baftizadeh F, Cossio P, Pietrucci F, Laio A (2012) Protein folding and ligand-enzyme binding from bias-exchange metadynamics simulations. Curr Phys Chem 2: 79–91.
65. Kumar S, Rosenberg JM, Bouzida D, Swendsen RH, Kollman PA (1992) The weighted histogram analysis method for free-energy calculations on biomolecules. i. the method. Journal of computational chemistry 13: 1011–1021.
