# Supplementary material for: Metadynamics Simulations Reveal a Na+ Independent Exiting Path of Galactose for the Inward-Facing Conformation of vSGLT
Source: PLoS Comput Biol. 2014 Dec 18;10(12):e1004017. doi: 10.1371/journal.pcbi.1004017 (PMC4270436; doi:10.1371/journal.pcbi.1004017)
Supplement: S3 Text — Mutant. (PDF) [file pcbi.1004017.s008.pdf]

Free Energy Profile of Y263F Mutant

Simulating the Y263F mutant, we can see how this mutation affects the free energy of the system while investigating the release of the two ligands. In Figure S3, we report the WT free energy profile along the path collective variable of Gal (black line) and that of the mutant Y263F (green line). We can clearly see that the profile is different, with the second minimum that becomes the global minimum, more stable and broader than the first one. This means that the mutation actually changes the stability of the configurations characterizing the exit profile of Gal. We remark that in the simulation of the mutant we have explored only the region between 1.2 and 4.5 of path collective variable. Concerning the structural features, we observe that Min1-Y263F is similar to Min1-WT. In Min1-Y263F Gal is parallel to F263, while in Min1-WT it can be parallel but also perpendicular to Y263. The coordination shell is very similar (H-bonds with E68, E88, Q428, N64). Min2-Y263F is characterized by different positions of Gal inside the binding site. Indeed, this minimum is quite broad and contains several different conformational states, among which also the one corresponding to Min2-WT. For this reason, also the residues coordinating Gal are different. It interacts with N260 but not with Q69. Other residues interacting with Gal are: E88, Y87, Q428. Moreover, as in Min2-WT (and at variance with Min1-Y263F), in Min2-Y263F Gal does not interact with E68.
